# Visually assessed severity of lumbar spinal canal stenosis is paradoxically associated with leg pain and objective walking ability

**DOI:** 10.1186/1471-2474-15-348

**Published:** 2014-10-16

**Authors:** Pekka Kuittinen, Petri Sipola, Tapani Saari, Timo Juhani Aalto, Sanna Sinikallio, Sakari Savolainen, Heikki Kröger, Veli Turunen, Ville Leinonen, Olavi Airaksinen

**Affiliations:** Department of Neurosurgery, Kuopio University Hospital, Puijonlaaksontie 2, PO Box 1777, Kuopio, 70210 Finland; Institute of Clinical Medicine, University of Eastern Finland, Kuopio, Finland; Department of Clinical Radiology, Kuopio University Hospital, Kuopio, Finland; Unit of Radiology, Institute of Clinical Medicine, University of Eastern Finland, Kuopio, Finland; Health Center Ikioma OY, Mikkeli, Finland; Institute of Public Health and Clinical Nutrition, University of Eastern Finland, Kuopio, Finland; Department of Orthopaedics and Traumatology, Kuopio University Hospital and Bone and Cartilage Research Unit, University of Eastern Finland, Kuopio, Finland; Department of Orthopaedics and Traumatology, Kuopio University Hospital, Kuopio, Finland; Unit of Neurosurgery, Institute of Clinical Medicine, University of Eastern Finland, Kuopio, Finland; Department of Physical and Rehabilitation Medicine, Kuopio University Hospital, Kuopio, Finland

**Keywords:** Spinal stenosis, Magnetic resonance imaging, MRI, Low back pain, Leg pain, Disability, Walking distance

## Abstract

**Background:**

Lumbar spinal stenosis (LSS) is the common term used to describe patients with symptoms related to the anatomical reduction of the lumbar spinal canal size. However, some subjects may have a markedly narrowed canal without any symptoms. This raises the question of what is the actual role of central canal stenosis in symptomatic patients. The purpose of this study was to compare radiological evaluations of LSS, both visually and quantitatively, with the clinical findings of patients with LSS.

**Methods:**

Eighty patients [mean age 63 (11) years, 44% male], with symptoms severe enough to indicate LSS surgery, were included in this prospective single-center study. Lumbar magnetic resonance imaging was performed and one experienced neuroradiologist classified patients into three groups: 0 = normal or mild stenosis, 1 = moderate stenosis, and 2 = severe stenosis. In addition, the same observer measured the minimal dural sac area level by level from the inferior aspect of L1 to the inferior aspect of S1. The association between radiological and clinical findings were tested with Oswestry Disability Index, overall visual analog pain scale, specific low back pain, specific leg pain, Beck Depression Inventory, and walking distance on treadmill exercise test.

**Results:**

In the visual classification of the central spinal canal, leg pain was significantly higher and walking distance achieved was shorter among patients with moderate central stenosis than in patients with severe central stenosis (7.33 (2.29) vs 5.80 (2.72); P = 0.008 and 421 (431) m vs 646 (436) m; P = 0.021, respectively). Patients with severe stenosis at only one level also achieved shorter walking distance than patients with severe stenosis of at least two levels. No correlation between visually or quantitatively assessed stenosis and other clinical findings was found.

**Conclusions:**

There is no straightforward association between the stenosis of dural sac and patient symptoms or functional capacity. These findings indicated that dural sac stenosis is not the single key element in the pathophysiology of LSS.

**Electronic supplementary material:**

The online version of this article (doi:10.1186/1471-2474-15-348) contains supplementary material, which is available to authorized users.

## Background

Lumbar spinal stenosis (LSS) is the term used commonly to describe patients with symptoms related to the anatomical reduction of the size of the lumbar spinal canal [1]. However, some subjects can have a narrowed canal without presenting any symptoms. Therefore, this peculiarity raised the question of what is the actually role of central canal stenosis in symptomatic patients. The relationship between radiological findings and patient’s symptoms has been studied by several authors. These studies have reported that MRI imaging findings did not identify symptomatic from asymptomatic persons [2–5].

Unfortunately, many previous studies have some methodological limitations related to the assessment of patients’ symptoms. Typically, the symptoms have been evaluated retrospectively from the patient records or otherwise rated without a standard methodology. The use of the standardized Oswestry Disability Index (ODI) [6], Visual analog pain scale (VAS) [7], Beck Depression Inventory (BDI) [8], and specific back pain at rest and leg pain at walking items of the Numerical Rating Scale (LBP- and LP-NRS-11) [9] have improved the accuracy and reproducibility of patients symptom and functional disability grading.

The purpose of this study was to investigate the role of anatomical changes on patient symptoms and functional disability. We compared the radiological findings to the symptoms and function of patients with LSS measured with standardized methods in a prospective study setting.

## Methods

### Patients

This prospective single-center study was approved by the Ethics Committee of Kuopio University Hospital, and the patients provided informed consent. The study included 84 patients with both clinically and radiologically defined LSS who had been selected for surgical treatment. Selection for surgery was made by an orthopedist or neurosurgeon at the Kuopio University Hospital, Kuopio, Finland. The inclusion criteria were as follows: 1) the presence of severe back, buttock, lower extremity pain, and/or neurogenic claudication with radiographic evidence (computed tomography, magnetic resonance imaging (MRI), myelography) of compression of the cauda equina or exiting nerve roots by degenerative changes (ligamentum flavum, facet joints, osteophytes, and/or disc material); and 2) clinical and radiological evaluation by a surgeon, indicating that the patient had degenerative LSS with symptoms that could be relieved by operative treatment. Additionally, all patients had a history of ineffective response to conservative treatment over three months. Patients with only back pain were not included.

The exclusion criteria were as follows: emergency or urgent spinal surgery precluding recruitment and protocol investigations; cognitive impairment prohibiting completion of the questionnaires or other failures in cooperation, and the presence of metallic particles in the body preventing the magnetic resonance imaging investigation. A previous spine operation or coexisting disc herniation was not an exclusion criterion, but the main diagnosis of the study patient had to be LSS. The surgeons sent the information of eligible patients to the Department of Physical and Rehabilitation Medicine, for further study organization. In four of these patients, recordings of standardized tests were not completed and these patients were excluded. Finally the study included 80 patients (mean age 63 ± 11 years, 44% male).

### MRI

MRI of the lumbar spine was performed with a 1.5-T imager (Vision; Siemens Medical Solutions, Erlangen, Germany) and a dedicated receive-only spine coil. All patients were evaluated prospectively by applying the same study protocol for study purposes. The imaging protocol conformed to the requirements of the American College of Radiology for the performance of MRI of the adult spine [10]. The following sequences were used: (a) sagittal T1-weighted spin-echo (repetition time/echo time (TR/TE) 600/12 ms; flip angle, 150°; 4-mm sections; intersection gap, 0.4 mm; field of view (FOV), 290 mm; rectangular FOV, 80%; three signals acquired per data line; matrix 288 × 512) *(b)* sagittal T2-weighted fast spin-echo (3500/120; flip angle, 180°; echo train length of five; 4-mm sections; intersection gap, 0.4 mm; FOV 290 mm; rectangular FOV, 63%; two signals acquired; matrix 180 × 512); *(c)* transverse T1-weighted spin-echo (700/15; flip angle, 90°; 4-mm sections; intersection gap, 0.4 mm; FOV, 250 mm; rectangular FOV, 80%; two signals acquired per data line; matrix 288 × 512); and *(d)* transverse T2-weighted fast spin-echo (5000/120; flip angle, 180°; echo train length of 15; 4-mm sections; intersection gap, 0.4 mm; FOV, 250 mm; rectangular FOV, 100%; three signals acquired per data line; matrix 330 × 512).

The entire lumbar spine was studied on the sagittal images (T12-S1), including parasagittal imaging of all the neural foramina bilaterally. Transverse images were obtained from the inferior aspect of L1 to the inferior aspect of S1, and the orientation of the sections was planned parallel to the major axis of each disc. In all sequences, a saturation band was placed over the abdominal vessels.

### Image analysis

**Figure 1:**
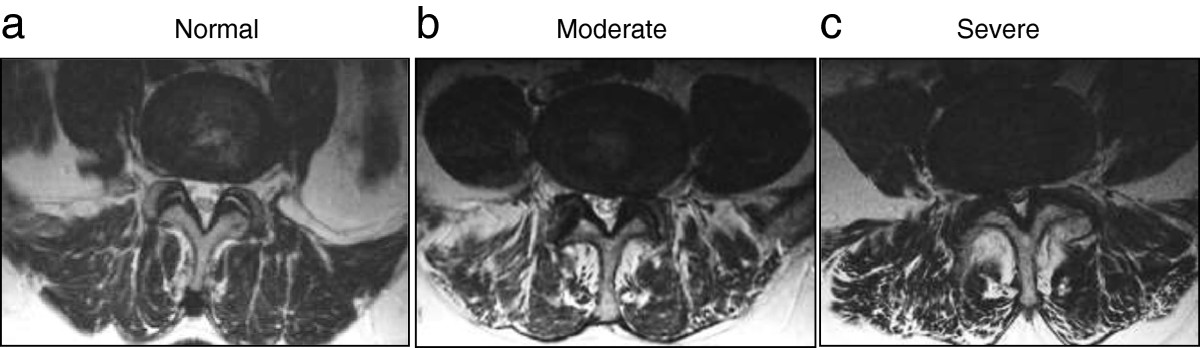
**Axial T2-weighted model images of representative cases that were used to grade central spinal canal in visual assessment. a)** normal central spinal canal; **b)** moderate central spinal canal stenosis; **c)** severe central spinal canal stenosis.

Image evaluation was performed with Numaris software (Siemens Medical Systems) by a neuroradiologist with 15 years of experience with spinal MRI (T.S.). Image analysis was performed independently without knowledge of the patient clinical symptoms and data. Each level from the inferior aspect of L1 to the inferior aspect of S1 was analyzed separately. For the visual image evaluation, the central canal was visually classified into three grades: 0 = normal or mild changes (ligamentum flavum hypertrophy and/or osteophytes and/or or disk bulging without narrowing of the central spinal canal), 1 = moderate stenosis (central spinal canal is narrowed but spinal fluid is still clearly visible between the nerve roots in the dural sac), 2 = severe stenosis (central spinal canal is narrowed and there is only a faint amount of spinal fluid or no fluid between the nerve roots in the dural sac) (Figure 1). Patients who had severe stenosis at least two levels in the visual analysis were classified as having the multilevel spinal stenosis. For the quantitative image evaluation, each level was first assessed visually. The borders of the dural sac were manually traced in the image with smallest cross-sectional area upon visual examination. According to the smallest area, patients were divided into three groups: 1) patients with dural sac area less than 75 mm^2^; 2) patients with dural sac area form 75–100 mm^2^; and 3) patients with dural sac area greater than 100 mm^2^[11]. In statistical analyses, the highest degree of stenosis was used for both the visually and quantitatively measured stenoses.

### Assessment of preoperative symptoms and functional disability

The overall current low back and leg pain intensity was assessed by a self-administered VAS (range 0–100 mm) in a sitting position during study visits. VAS was proven as a valid index for experimental and clinical assessments of chronic pain [12]. According to the score range (0–100 range), four groups were established: scores 0–20 (minimal), 21–40 (moderate), 41–60 (severe), and over 60 (crippled). Back pain at rest (during last week) and leg pain while walking (during last week) were measured separately with a numeric rating scale ranging from 0 to 10 (NRS-11) [9]. The questions about pain were anchored on the left (0) with the phrase “No pain” and on the right (10) with the phrase “intolerable pain”.

Subjective disability was measured by the validated Finnish version of the ODI, where 0% represents no disability and 100% extreme debilitating disability. This ODI score (0–100 range) were also classified in four groups: scores 0–20 (minimal), 21–40 (moderate), 41–60 (severe), and over 60 (crippled) [3, 6, 13].

Depression was assessed with the Finnish version of the 21-item BDI with scores ranging from 0–63 [8, 14]. The cutoff point for depression was set at 15/63. The BDI score was classified into two groups: scores 0–14 (normal mood), and 15 or more (indicating elevated depressive symptoms) [15].

The treadmill test was supervised by a physiotherapist. The patient was asked to keep a straight, upright position during walking on ram without elevation. The starting speed was 0.67 m/s for the first 10 min (400 m), then 1 m/s for the next 10 min (600 m), with a maximum result 1,000 m in 20 min. If the patient was not able to start with a speed of 0.67 m/s, another test with a starting speed of 0.5 m/s was applied. The walking distance scale ranged from 0 to 1000 m.

### Statistical analyses

Associations between the quantitative evaluation of the radiological stenosis in MRI and the continuous ODI, VAS, BDI, and walking capacity were analyzed using Spearman correlation coefficients. The visual assessments were analyzed using *t*-test. Non-parametric tests were used when no assumption of normal distribution could be made. Statistical analysis was performed using SPSS for Windows (version 19.0; SPSS, IBM, Chicago IL, USA). Statistical significance was set at a P <0.05.

## Results

### Clinical characteristics, preoperative symptoms, and functional disability

Patient characteristics are summarized in Table 1. The mean age of the study patients (n = 80) at the time of surgery was 63 years (11) [mean (SD)], and 35 (44%) of the subjects were male. Ten patients (13%) had undergone previous spine operation. Coexisting disc herniation was found in 11 patients (14%). According to the ODI scores 7 (9%), 26 (33%), and 36 (45%) had minimal, moderate, and severe disability, respectively, and 11 (14%) patients were crippled. Regarding the overall VAS scores, 28 (35%) patients had minimal pain, 20 (25%) had moderate pain, 22 (28%) had severe pain, and 10 (13%) had crippled pain. Regarding the BDI scale, 63 patients had normal mood and 17 patients were depressed (15 or more points); the mean BDI was 10.4 (6.1). Mean walking distance achieved was 545 (445) m (range, 0–1000 m).

**Table 1:** **Clinical characteristics of the study subjects**

	All patients (n = 80)	Moderate stenosis (n = 36)	Severe stenosis (n = 44)	P-value
Male/female	35/45 (43/56)	18/18 (50/50)	17/27 (39/61)	0.308
Marital status married or co-habiting	52 (65)	25 (69.5)	27 (61.3)	0.762
Current smoker	17 (21.3)	10 (27.7)	7 (16.0)	0.345
Previous lumbar operation	11 (13)	6 (16.7)	5 (11.4)	0.248
Depressed^ȝ^	17 (21)	6 (16.7)	11 (25.0)	0.365
Minimal dural sac area^ɫ^	56.1 (21.9)	72.1 (18.0)	43.0 (15.2)	0.000
Age	63.0 (11.0)	62.0 (11.6)	64.0 (10.8)	0.413
BMI (kg/m^2^)	29.6 (4.0)	29.5 (4.4)	29.8 (3.5)	0.746
Number of somatic diseases	5.3 (3.0)	5.6 (3.2)	5.1 (2.8)	0.445
ODI	44.7 (16.1)	45.4 (18.0)	44.1 (14.4)	0.731
VAS overall	33.3 (23.9)	34.6 (24.7)	31.9 (25.0)	0.523
NRS LBP	4.1 (2.6)	4.72 (2.6)	3.59 (2.4)	0.059
NRS LP	6.49 (2.6)	7.33 (2.29)	5.80 (2.72)	0.008
BDI score	10.4 (6.1)	9.78 (6.86)	10.93 (5.546)	0.233
Walking distance (m)	545 (445)	421 (431)	646 (436)	0.021
Foraminal stenosis (normal/moderate/severe)^§^		53/39/8	55/36/9	0.971
Multilevel stenosis yes/no^§^		53/47	70/30	0.104
Lumbar fusion yes^§^		33	66	0.258

Note: Except where indicated, data are numbers of patients, with percentages in parentheses or means, ± standard deviations in parentheses.

^ȝ^BDI 15 or more points, ^ɫ^at the most stenotic level, mean (mm^2^), ± standard deviations in parentheses. ODI = Oswestry Disability Index (0–100), VAS overall = Visual analog pain scale (0–100), NRS LBP = Numerical rating scale low back pain at rest, scale (0–10), NRS LP = Numerical rating scale leg pain at walking, scale (0–10), BDI = Beck Depression Inventory (0–63), ^§^Percentages.

### Radiological findings

According to the visual assessment, none of the patients had a normal central canal. The central canal was moderately and severely stenosed in 36 (45%) and 44 (55%) patients, respectively. Based on the quantitative assessment, the mean minimal dural sac area was 56.1 (21.9) (range, 12–120) mm^2^. In the quantitative analyses the smallest dural sac was greater than 100 (75–100) mm^2^, and under 75 mm^2^ in 4 (5%), 15 (19%), and 61 (76%) patients, respectively.

### Correlation of imaging findings with preoperative symptoms and functional disability

**Figure 2:**
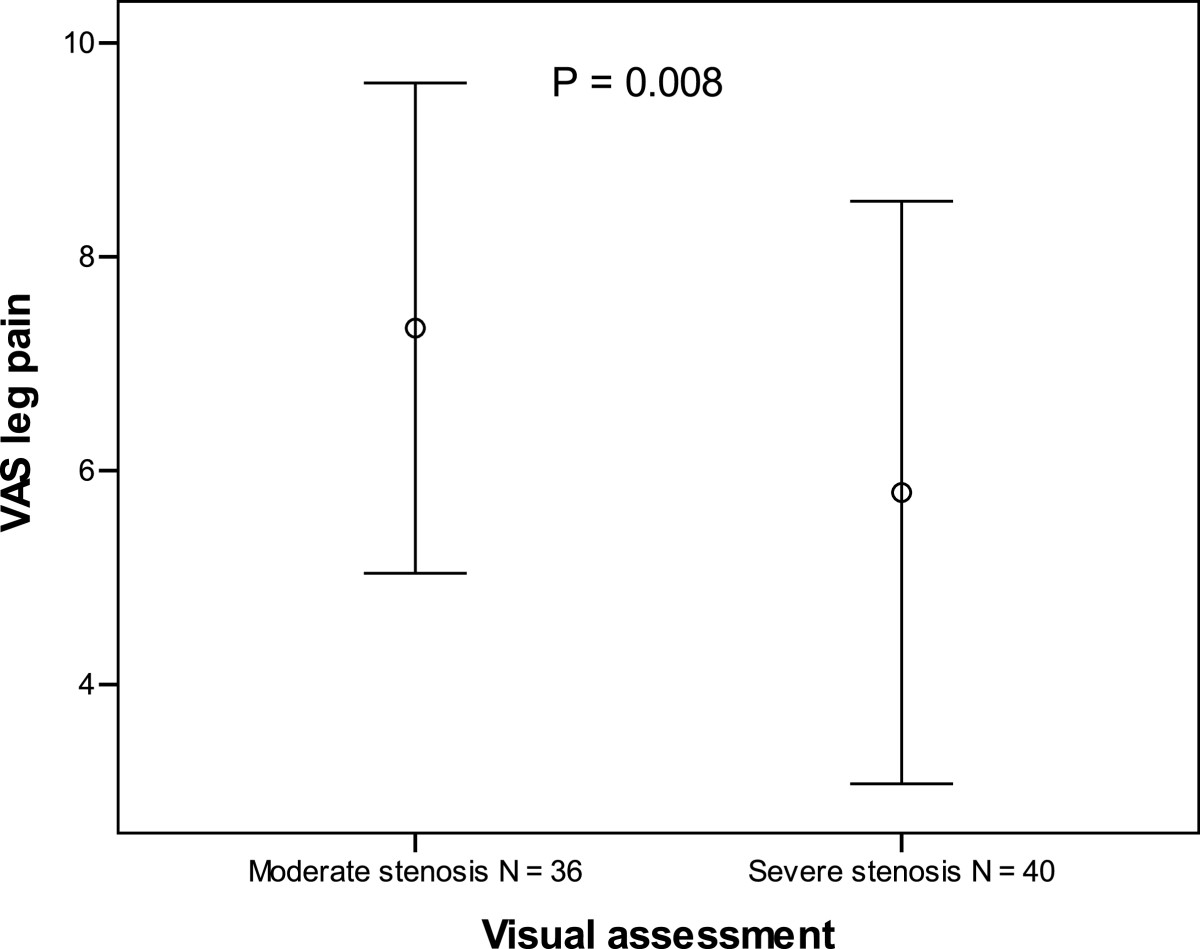
**Visual assessment of the MRI and VAS leg pain n = 80.** Mean visual analog scale (VAS) leg pain ±1 standard deviation.

**Figure 3:**
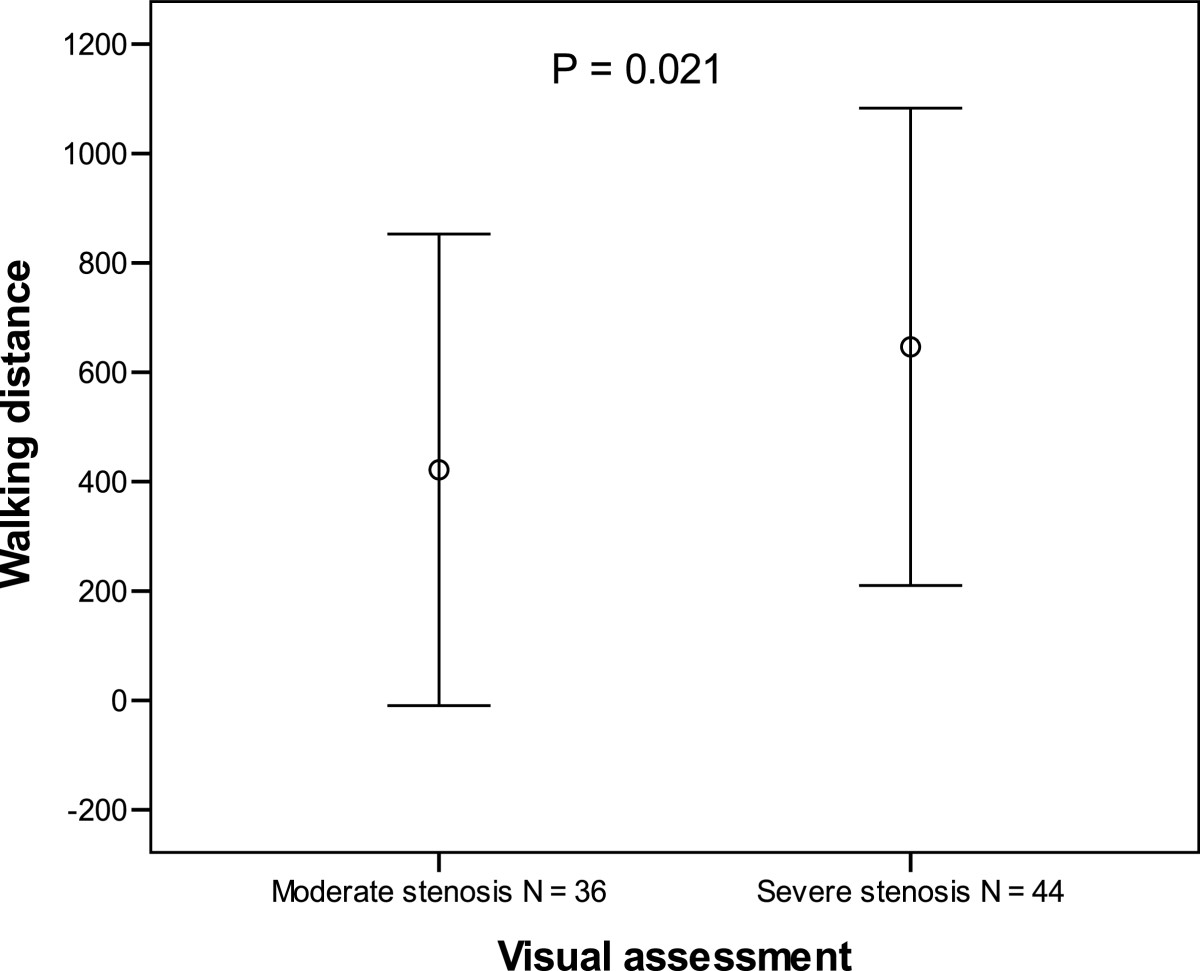
**Walking distance in the treadmill exercise test in patients with moderate and severe spinal stenosis.** Mean walking distance ±1 standard deviation.

The correlation of radiological spinal stenosis and clinical symptoms is summarized in Table 1. VAS leg pain was higher in patients with moderate stenosis than in patients with severe stenosis (7.33 (2.29) vs 5.80 (2.72); *P* = 0.008) (Figure 2). The walking distance achieved was shorter in patients with radiologically moderate stenosis than in patients with severe stenosis (421 (431) m vs 646 (436) m; P = 0.021) (Figure 3). Patients with severe stenosis at only one level (50%) achieved shorter walking distance than patients with severe stenosis of at least two levels [393 (436) m vs 675 (423) m; *P* = 0.022]. No correlation was found between the dural sac area measurements and patient symptoms or walking distances achieved (Table 2).

**Table 2:** **Correlation of the dural sac area with patient symptoms and walking capacity**

	ODI scale	VAS overall scale	NRS LBP	NRS LP	Beck depression index	Treadmill test
Central spinal canal (mm2)	r = 0.086	r = -0.036	r = -0.004	r = 0.023	r = -0.099	r = -0.145
	P = 0.448	P = 0.752	P = 0.972	P = 0.841	P = 0.380	P = 0.200

r = Spearman correlation coefficients.

P = P values.

ODI = Oswestry Disability Index (0–100).

VAS overall = Visual analog pain scale (0–100).

NRS LBP = Numerical rating scale low back pain at rest (0–10).

NRS LP = Numerical rating scale leg pain at walking (0–10).

Treadmill exercise test (0–1000 m).

BDI = Beck Depression Inventory (0–63).

## Discussion

The strength of this study was its prospective study design for both radiological and clinical methods. The main finding of our study was that there is no linear correlation in the radiological degree of severity of LSS and clinical findings. In contrast, according to the visual evaluations of the central canal LSS, leg pain measured by VAS was higher in the moderate stenosis group than in severe stenosis group. Additionally, the walking distance achieved was shorter in the patients with moderate stenosis on visual evaluation compared with the patients with severe stenosis. This finding was consistent for both the analysis performed using the maximal degree of stenosis and among patients with multilevel stenosis. We did not find any correlation between objective quantitative radiological measures and patient symptoms, which also supports the paradoxical finding based on visual evaluation.

Our results indicate that LSS is not solely an anatomical disorder, but that this disease may have other underlying pathobiological mechanisms to be discovered. Indeed, we found that the correlation between the severity of LSS and clinical findings is complex, with milder symptoms in patients with more severe stenosis. In a cross-sectional study setting, we cannot provide a definite explanation for our findings. Our results raise the possibility that the pain of patients could resolve spontaneously across time and that this adaptation could possibly explain the longer walking distance achieved in this group of patients regardless of the progression in the severity of the central LSS. One possible explanation to our apparently paradoxical findings could be the decreased lumbar spine instability in patients with advanced facet joint hypertrophy and large end-plate osteophytes, which in turn would provide pain relief and allow higher walking capacity. Accordingly, degenerative hypertrophy could be a protective mechanism against the disc degeneration typically found in patients with advanced age. Porter and Ward hypothesized that central stenosis at two levels or central stenosis at one level with lover root canal stenosis may cause venous congestions and may explain neurogenic claudication [16]. We are not aware of the methods to assess venous congestion on the MRI, which could be a potential target of future research.

Sirvanci et al. found no correlation between the severity of spinal stenosis and ODI. The aforementioned study, however, was retrospective and patient symptoms were evaluated only by the ODI scale. Moreover, no data of the experience of the subjects performing the radiological analysis were provided in that study [5]. Accordingly, it is difficult to evaluate the reliability of the radiological analysis. In the study by Geisser et al., no correlation was found between the quantitative measurement of central spinal canal AP diameter and clinical symptoms [3]. Assessment of spinal canal AP diameter may be problematic in the context of LSS because, according to our experience, the most common reason for LSS is facet joint hypertrophy that causes bilateral stenosis of the dural sac and does not influence the mid-sagittal level. Jonsson et al. found a weak positive correlation between the central spinal canal AP diameter and reduction of the patient’s estimated walking ability; however, that correlation was not statistically significant [4]. Haig et al. evaluated the LSS by measuring the area of the minimal dural sac cross-sectional area, as also performed in our study, and they found no difference in the degree of stenosis between symptomatic and asymptomatic subjects [2]. Interestingly, in the one other study using validated methods to record patient symptoms, patients with multilevel spinal stenosis had significantly better scores in the general health items of the Short Form-36, and similar to our findings, moderate lower leg pain measured with the VAS-scale [17]. Further, Park et al. found that there was less pain radiation and pseudoclaudication in patients with three- and two-level spinal stenosis compared with patients with one-level stenosis only [18]. In contrast, Ogikubo et al. found lowered preoperative walking capacity, higher leg and back pain and reduced quality of life in LSS patients with smaller dural sac cross-sectional area [19]. Furthermore, Yukawa et al. found a positive correlation between the preoperative dural cross-sectional area in magnetic resonance imaging and with a better postoperative ODI score [20]. The aforementioned and many other studies [2–5, 17] did not analyze spinal canal stenosis visually, which we considered an elemental part of the image analysis, especially in patients with stenosis at the upper part of the lumbar spine. The amount of neural tissue at L1-2 and L2-3 levels is considerably greater than at the L4-5 or at the presacral measurements, and thus, by performing dural sac cross-sectional area measurements only, subjects with reduced space for neural tissue may not be correctly recognized.

We did not execute intra-rater and inter-rater repeatability of MRI evaluation, and this is the main weakness of this study. However, we consider that such a measurement was not related to the present study aim. Notably, reliability of the qualitative grading of LSS has been described and evaluated previously, and it was shown to have substantial intraobserver and moderate interobserver agreement in a multicenter study setting. In the present study methods, a 7-grade classification has been used [21]. However, Lurie et al. used a 4-grade classification in their study and showed moderate to substantial reliability [22]. Moreover, we have recently extended the method of the assessment of lateral stenosis using a 3-grade classification, which has been demonstrated to have acceptable repeatability for research purposes [23]. Future objective would be standardized studies of visual assessment of the LSS and to analyze how these findings correlate with patient symptoms and surgical outcomes.

The results of the current study relate to routine clinical MRI with patients lying in the supine position. Imaging studies of patients in this position is a limitation because patient symptoms may worsen in an upright position. Further, the anatomy of the neural canal may appear altered when patients are in an upright position. Accordingly, the upright position would be the most appropriate imaging acquisition posture to link imaging findings to patient symptoms [24–27]. Hiwatashi et al. found that axial loading while performing imaging studies could even influence to treatment decisions [27].

The incidence of LSS is increasing probably because of the better quality and availability of radiological imaging equipment, and facilities, added to increasing aging population [28, 29], which reflect in a higher number of LSS surgery. However, selection of patients for surgical treatment still remains challenging. Our results strengthen the classical conception that the diagnosis of this syndrome is constituted by the clinical history, clinical symptoms and radiographic evidence of a demonstrable stenosis [30–32].

MRI evaluations are thus needed to establish the level (s) and severity of stenosis. However, MRI images cannot be the only decision-making factor of surgical treatment selection for LSS patients. The degree of the severity of the disease cannot be judged based solely on MRI either. Ohtori et al. found that proinflammatory cytokine levels in the cerebrospinal fluid of patients with LSS correlated with the severity of the stenosis [33]. Sairyo et al. found that hypertrophy of the lumbar ligamentum flavum is associated with inflammation-related genes [34]. Moon et al. pointed out that fibrosis and scarring during inflammatory reaction is the major pathomechanism of ligamentum flavum hypertrophy [35]. The present study adds to the current knowledge by showing that there is no straightforward association between stenosis of dural sac and patient symptoms or functional capacity, which indicates that dural sac stenosis is not the only key in the pathophysiology of LSS. It is not justified to select patients for surgery based solely on the degree of central stenosis either.

## Conclusions

Association between the anatomical degree of LSS and the clinical findings is a complex one. Our findings indicate that advanced degenerative hypertrophy may potentially be a protective mechanism that causes relief of patient symptoms. Follow-up studies are needed to confirm if symptoms of patients with LSS may improve despite the progression of the anatomical degree of central LSS.

## Authors’ original submitted files for images

Below are the links to the authors’ original submitted files for images. Authors’ original file for figure 1 Authors’ original file for figure 2 Authors’ original file for figure 3
