# Supplementary figures and images for: Visually assessed severity of lumbar spinal canal stenosis is paradoxically associated with leg pain and objective walking ability
Source: BMC Musculoskelet Disord. 2014 Oct 16;15:348. doi: 10.1186/1471-2474-15-348 (PMC4203914; doi:10.1186/1471-2474-15-348)

**a****Normal**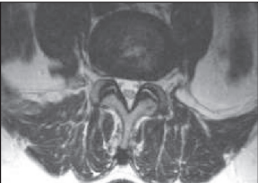**b****Moderate**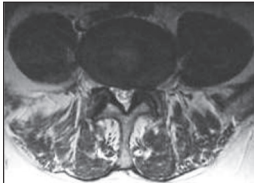**c****Severe**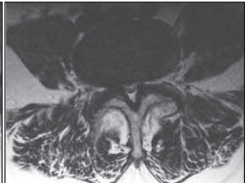

Supplement: Supplementary file 1 — Authors’ original file for figure 1 [file 12891_2013_2281_MOESM1_ESM.pdf]

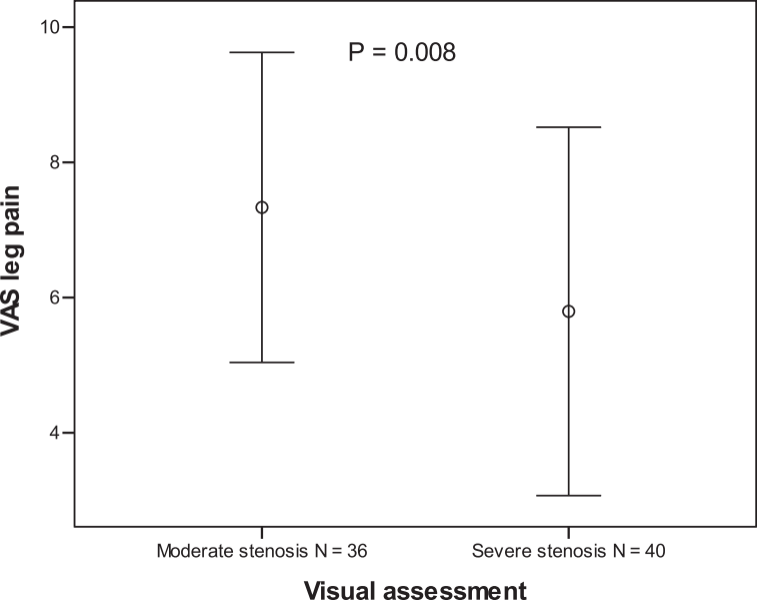

Supplement: Supplementary file 2 — Authors’ original file for figure 2 [file 12891_2013_2281_MOESM2_ESM.pdf]

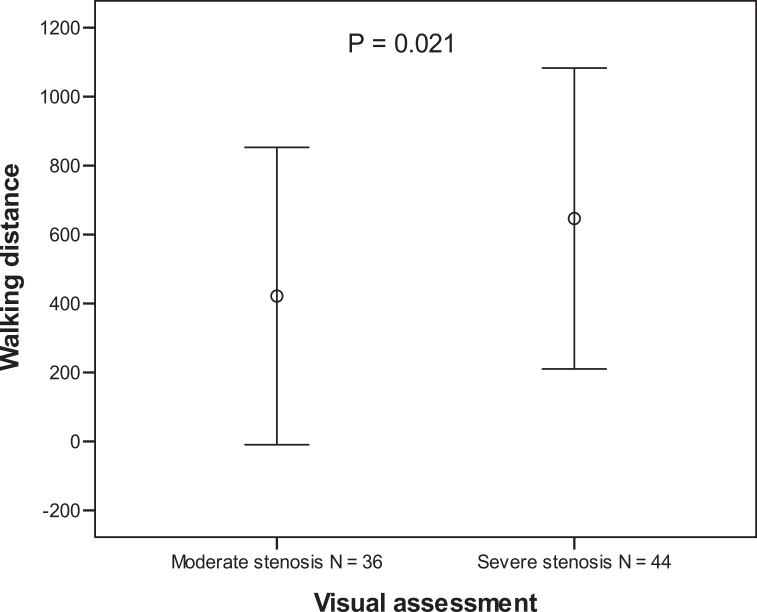

Supplement: Supplementary file 3 — Authors’ original file for figure 3 [file 12891_2013_2281_MOESM3_ESM.pdf]
